# Stouffer’s Test in a Large Scale Simultaneous Hypothesis Testing

**DOI:** 10.1371/journal.pone.0063290

**Published:** 2013-05-14

**Authors:** Sang Cheol Kim, Seul Ji Lee, Won Jun Lee, Young Na Yum, Joo Hwan Kim, Soojung Sohn, Jeong Hill Park, Jeongmi Lee, Johan Lim, Sung Won Kwon

**Affiliations:** 1 Korean Bioinformation Center, Korea Research Institute of Bioscience and Biotechnology, Daejeon, Republic of Korea; 2 College of Pharmacy, Seoul National University, Seoul, Republic of Korea; 3 Toxicological Evaluation and Research Department, National Institute of Food and Drug Safety Evaluation, Korea Food and Drug Administration, Seoul, Republic of Korea; 4 School of Pharmacy, Sungkyunkwan University, Suwon, Republic of Korea; 5 Department of Statistics, Seoul National University, Seoul, Republic of Korea; New Jersey Institute of Technology, United States of America

## Abstract

In microarray data analysis, we are often required to combine several dependent partial test results. To overcome this, many suggestions have been made in previous literature; Tippett’s test and Fisher’s omnibus test are most popular. Both tests have known null distributions when the partial tests are independent. However, for dependent tests, their (even, asymptotic) null distributions are unknown and additional numerical procedures are required. In this paper, we revisited Stouffer’s test based on z-scores and showed its advantage over the two aforementioned methods in the analysis of large-scale microarray data. The combined statistic in Stouffer’s test has a normal distribution with mean 0 from the normality of the z-scores. Its variance can be estimated from the scores of genes in the experiment without an additional numerical procedure. We numerically compared the errors of Stouffer’s test and the two p-value based methods, Tippett’s test and Fisher’s omnibus test. We also analyzed our microarray data to find differentially expressed genes by non-genotoxic and genotoxic carcinogen compounds. Both numerical study and the real application showed that Stouffer’s test performed better than Tippett’s method and Fisher’s omnibus method with additional permutation steps.

## Introduction

We frequently encounter complex hypothesis testing problems that require the combination of several independent or dependent test results. For example, in our experiment motivating this work, we wanted to assess the pharmacological effects of carcinogens on gene expression levels. As carcinogens can be classified as genotoxic and non-genotoxic, depending on the mechanism of action, we wanted to understand the total mechanism of carcinogen action through the comparison of gene expression patterns for these two groups. The experiments were individually conducted for 3 genotoxic carcinogens (2-AAF, 3′MeDAB and DEN) and 3 non-genotoxic carcinogens (clofibrate, DL-ethionine and 1,4-dioxane). Both had their own control group and the experiments were repeated three times. In both genotoxic and non-genotoxic experiments, we tested the expression levels of each compound and the common control. We thereby obtained three p-values, which are dependent on each other by sharing the common control group. In this example, the test for each compound is denoted as a *partial test* for the hypothesis on expression levels of the compound. The null hypothesis for the pharmacological effects of carcinogens is written as the intersection of three hypotheses for three compounds, which is denoted as *a complex hypothesis*.

A lot of previous literature has reported the combination of partial tests of a complex hypothesis [1]. Below, we have listed a few commonly used combining functions. Suppose we have 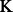 partial tests for the 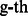 gene and their p-values are 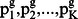. Tippett (1931) [2] proposed to use
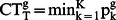
(1)whose null distribution, if the 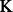
partial tests are independent and continuous, is the minimum of 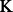 independent uniform random variables on 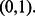 For a dependent partial test, it allows for bounds on the rejection probability according to the Bonferroni inequality. Fisher (1932) [3] proposed to use
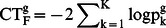
(2)which is often called Fisher’s omnibus test and Fisher’s omnibus function. It is well known that if the 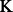
partial test statistics are independent and continuous, then the null distribution of 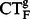 follows a central 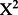 distribution with 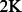 degrees of freedom. Stouffer (1949) [4] and Liptak (1958) [5] proposed to use
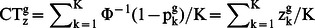
(3)where 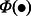 is the standard normal cumulative distribution function. Again, if the 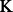 partial test statistics are independent and continuous, then its null distribution is normally distributed with mean 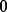 and variance 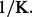 The classical methods are well reviewed by Owen (2009) [6].

Unlike the case of independent partial test statistics, the exact distribution of the combined statistics are unknown if they are dependent to each other. The most common remedy to dependent partial test statistics is to approximate their null distributions using additional resampling-based procedure. The permutation procedure is the most common additional procedure to get the null distribution of the combined tests. However, when applied to the microarray data analysis, it has at least two shortcomings. First, the microarray data analysis commonly tests a huge number of genes simultaneously, in which the test of each gene is based on combining several dependent partial tests; this arises in our motivating example, which will be introduced in the section “Materials and Model”. Thus, it is computationally heavy. Second, the multiple testing procedure in microarray data analysis often makes a decision among small p-values; the thresholding value to find DEGs is quite small. Thus, the required number of permutations should also be very large. For example, to approximate well the true p-value 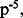 the number of permutation samples should be much larger than 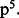 This, along with the first shortcoming, makes the resampling-based procedure inconvenient for large-scale microarray data analysis (see Westfall and Young (1993) [7] and references therein).

Some theoretical approximations to the null distribution are also reported in the literature. Brown (1975) [8] assumes that the partial statistics are from multivariate normal distribution with known covariance matrix, and develops an approximation to the null distribution of Fisher’s statistic 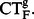 Kost and McDermott (2002) [9] extend the Brown’s approximation to the partial statistics, which are distributed as multivariate t-distribution with common denominator. Recently, Yang (2010) [10] thoroughly compare existing approximations to the null distribution of Fisher’s statistic, which include the aforementioned two approximations and that based on permutations.

Despite much interest, all existing approximations on dependent partial test statistics are focused on single hypothesis testing, and their applications to multiple hypothesis testing are not studied much. In multiple testing problem, many replications of partial test statistics are available and their dependent structure (covariance matrix) can be estimated from data. For example, in microarray data to find differentially expressed genes (DEGs), we would compute a set of partial test statistics for each gene, and have many sets of partial test statistics; the number of sets is equal to the number of genes in the data.

In this paper, we propose to estimate dependence structure (covariance matrix) of partial test statistics from this replication over genes. In particular, we are interested in 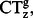 Stouffer’s test, which, in theory, is normally distributed with mean 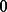 and unknown variance even for “dependent partial test statistics”. Thus, the dependence of partial test statistics can simply be estimated by estimating their covariance matrix. Our newly proposed procedure can be interpreted as a data dependent version of Brown (1975) [8] and Kost and McDermott (2002) [9]. They make theoretical derivation (approximation) on null distribution of combined test statistic under strong distributional assumption to the data. On other hand, we assume the covariance matrix of partial test statistics are same over genes, and estimate it from observed statistics. The null distribution of 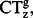 Stouffer’s test statistic, is evaluated as the normal distribution with mean 0 and the variance from the estimated covariance matrix.

We proposed a two-step procedure to estimate 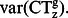 In the first step, we conservatively chose the number of true null genes by plotting the histogram of 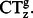 We chose genes that satisfied 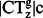 for an appropriately chosen 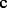 to guarantee the selected genes are surely equally expressed. Details on the choice of 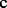 are followed in the section “Combined test using z-scores”. In the second step, we estimated the common covariance matrices of 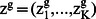 from pre-detected null genes in the first step, where 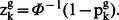 The first step might not be required if the proportion of null gene was close to one.

In this study, we describe (1) the preparation of samples that motivated the experiment, (2) the procedure to find differentially expressed genes (DEGs) using Stouffer’s z-score based method, (3) the numerical comparison of the receiver operating characteristic (ROC) curves of the Stouffer’s test 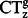 and two p-value based procedure, 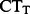 and 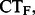 and (4) the investigation of differentially expressed genes (DEGs) between (i) control and non-genotoxic compounds, and (ii) control and genotoxic compounds.

## Materials and Models

### Ethics Statement

All animal procedures were approved by the Institutional Animal Care and Use Committee of NIFDS (0901KFDA029).

### Microarray Design

**Figure 1:**
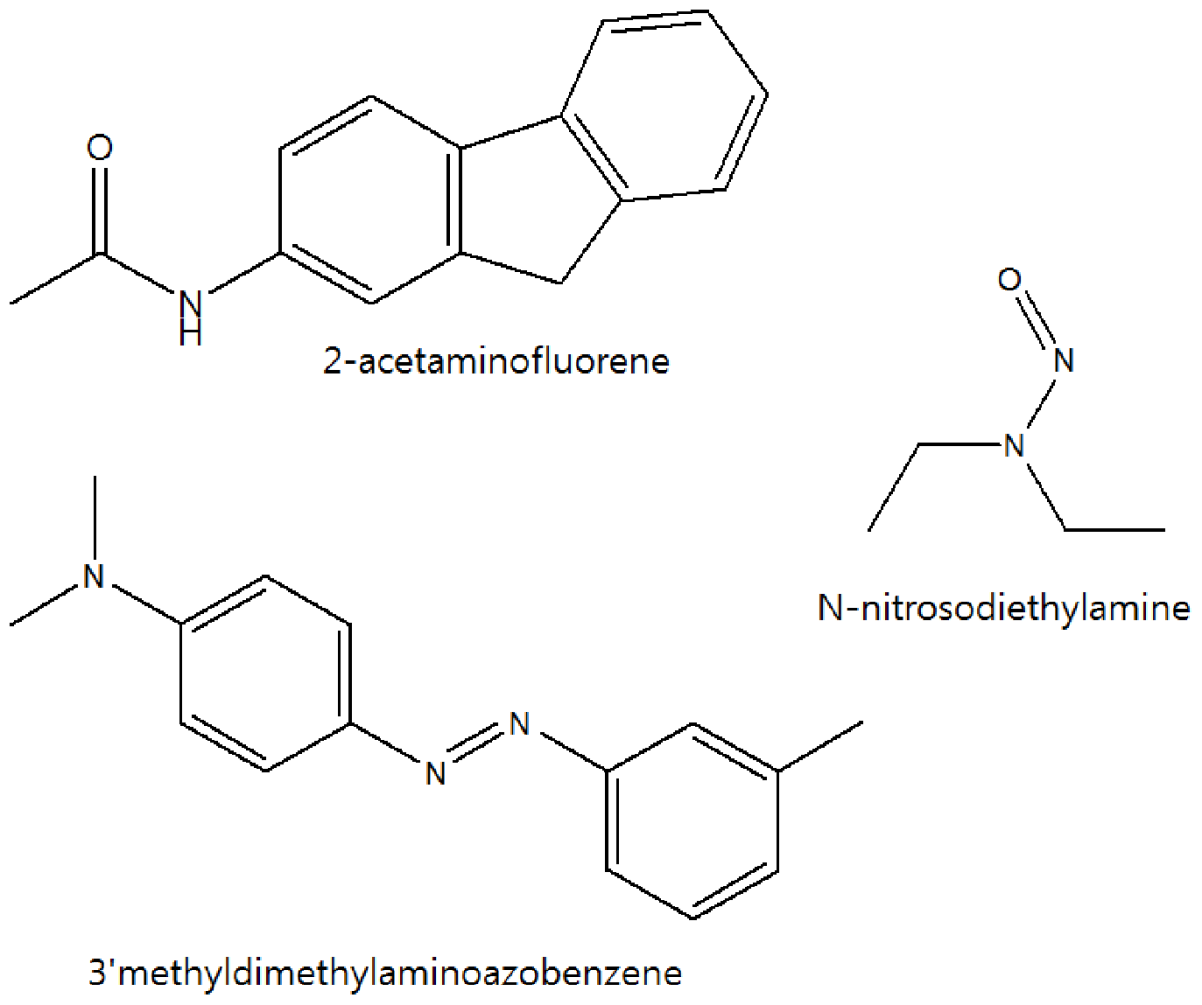
Structures of three genotoxic compounds.

**Figure 2:**
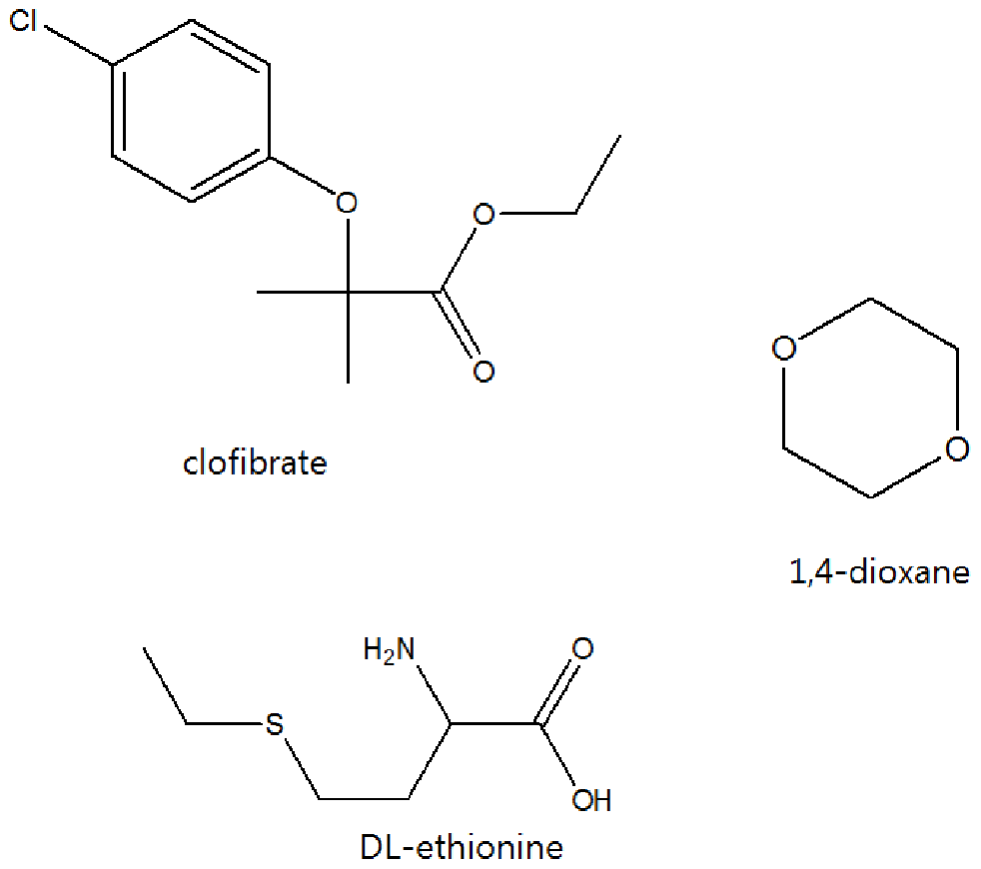
Structures of three non-genotoxic compounds.

We conducted a series of microarray experiments to understand carcinogenicity of compounds in view of toxicogenomics. There were six target compounds: 2-acetaminofluorene (2-AAF), 3′methyldimethylaminoazobenzene (3′MeDAB), N-nitrosodiethylamin (DEN), clofibrate, DL-ethionine and 1,4-dioxane, which are well-known carcinogens. Among them, 2-AAF, 3′MeDAB and DEN have genotoxicity; it was reported that 2-AAF and 3′MeDAB bind to DNA and cause hepatocarcinogenesis by DNA adduct formation [11] [12], while DEN has genotoxicity since a mutagenic effect was observed in the comet assay [13]. Clofibrate, DL-ethionine and 1,4-dioxane are classified as non-genotoxic carcinogens. It was reported that clofibrate, a peroxisome proliferator, stimulates the peroxisomal fatty acid beta-oxidation system and accordingly causes non-genotoxic cancer [14]. DL-ethionine was related to intrachromosomal recombination [15], and 1,4-dioxane was classified as a non-genotoxic since it had no activity in DNA repair assay [16]. Each compound dissolved in vehicle control was administered into rats. Once toxicity appeared, microarray was performed by using livers taken from the rats. To adjust errors in animal study, each compound was repeatedly administered into three individual rats (table 1). Only vehicle control was administered into three individual rats to produce control group. The structures of the three genotoxic and three non-genotoxic compounds are shown in Figure 1 and 2. And control was corn oil. The data sets are available from GEO, GSE31307.

**Table 1:** The design of the microarray experiments.

Genotoxic Carcinogen	Control	2-AAF	3′MeDAB	DEN
# of replicates	3	3	3	3
Non-genotoxic Carcinogen	Control	clofibrate	DL-ethionine	1,4-dioxane
# of replicates	3	3	3	3

### Statistics Design

The experiments for genotoxic and non-genotoxic agents are designed to have four arms: one arm for the control and three arms for the carcinogenic compounds. Each arm has three replicates that record the expression levels of 30,199 genes. The main goal of the study was to find a list of genes that show differential expression between **(A1)** control versus genotoxic carcinogen, **(A2)** control versus non-genotoxic carcinogen. To do this, we tested the hypothesis for the 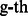 gene with 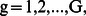 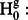 the expression level of the 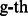
gene is not differentially expressed. Thereby, three partial test results for three genotoxic (or non-genotoxic) compounds were obtained and the results were combined to test the hypothesis 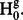


## Results and Discussion

### Combined Test using z-scores

We recalled the hypothesis for the 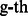 gene 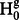 that the 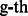
gene is not differentially expressed between two comparison groups, the control versus each of the genotoxic (or non-genotoxic) compounds. As stated in the experimental section, we had three partial tests for three compounds, 2-AAF, 3′MeDAB, and DEN (or clofibrate, ethionine, and 1,4-dioxane). We let 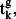 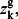 and 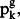 for, 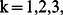 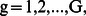 be their testing statistics, z-scores, and p-values.

First, let us consider the tests for the aims (A1) and (A2) in the experimental section. For these goals, Tippett’s test
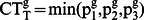
and Fisher’s omnibus test



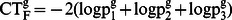
would be the two most common combining functions to test 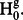 The reference distributions for both are well understood when test statistics 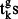 are independent of each other. However, in our example, test statistics of partial tests share a common control group, and they are dependent on each other. Thus, null distributions of both 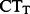
and 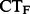
are not available analytically.

The re-sampling-based procedures, or permutation procedure more specifically, are commonly used to approximate the null distributions of 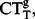 and 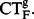 In this paper, we proposed to use z-score rather than p-value. The z-score for gene 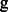 is defined as
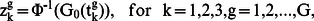
where 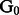 is the cumulative distribution function (CDF) of the test statistics 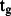 under the null (A1) or (A2). Here, 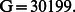 Under the null, it has the standard normal distribution. We proposed to use Stouffer’s statistic



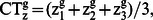
and, under the null, it has a normal distribution with mean 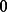 and variance
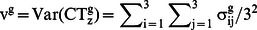
(4)where 
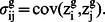
 In practice, 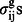 are unknown but can be estimated from the replicates of 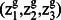 over genes under the assumption that they are equal over equally expressed genes.

Now we introduce the two-step procedure to estimate the null distribution. In the first step, we select the null genes by plotting the histograms of 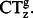 We select the genes with 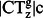 for an appropriately chosen 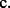 The thresholding value 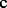 is chosen to satisfy “zero assumption” by Efron (2004) [17], which is also termed “purity” by Genovese and Wasserman (2004) [18]. The details on the choice of 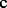 is very same with that in Efron (2004) [17]. We then estimate the covariance matrices of 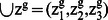 using the sample covariance matrixes of the selected genes. To be specific,
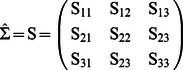
where 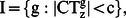 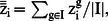 and



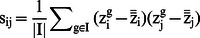



for 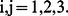 Finally, the variance of 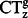 is estimated by
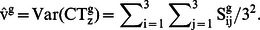



In summary, the procedure of this paper is:

S1) Find the z-scores 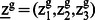 for 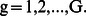


S2) Compute the Stouffer’s test statistic 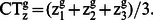


S3) Find the set 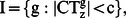 for 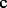 satisfying Efron’s zero assumption.

S4) Compute the estimate of variance of 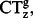 which is notated as 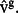


S5) Test the expression levels of gene g, using statistic 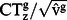 and standard normal distribution.

### Numerical Study

We numerically compared the powers of Stouffer's test, Tippett’s test and Fisher’s omnibus test. In comparison, we additionally consider Dunnett’s test (Dunnett (1955) [19]), which tests the difference between multiple treatment groups to a single control group. The p-values of Tippett’s test and Fisher’s omnibus test were approximated by additional permutation procedures.

For the study, we generated data sets that had the same structure (or dimension) as that of our motivating example. To be specific, we had one control group and three compound (agent) groups and changed the number replicates in each group to 5 and 20. We fixed the total number of genes at 4,000, and the number of differentially expressed genes (DEGs) at 200. Thus, the number of equally expressed genes (EEGs) was 3,800. Let 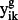 be the expression level of the 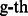 gene for the 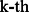 replicates of the 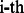 compound. Here, 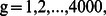 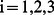 and 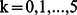 (or 20); 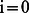 indicated the control group. We assumed that, for the control group, 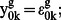 for the compound groups 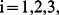


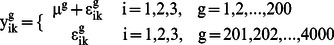
where 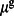 is fixed as 1.5, 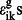 are independently and identically distributed (IID) from the normal distribution with mean 0 and variance 1; the t-distribution with 5 degrees of freedom (dfs); and the gamma distribution with parameters 3 and 1. We used 5,000 permuted samples in doing 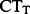 and 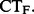


**Figure 3:**
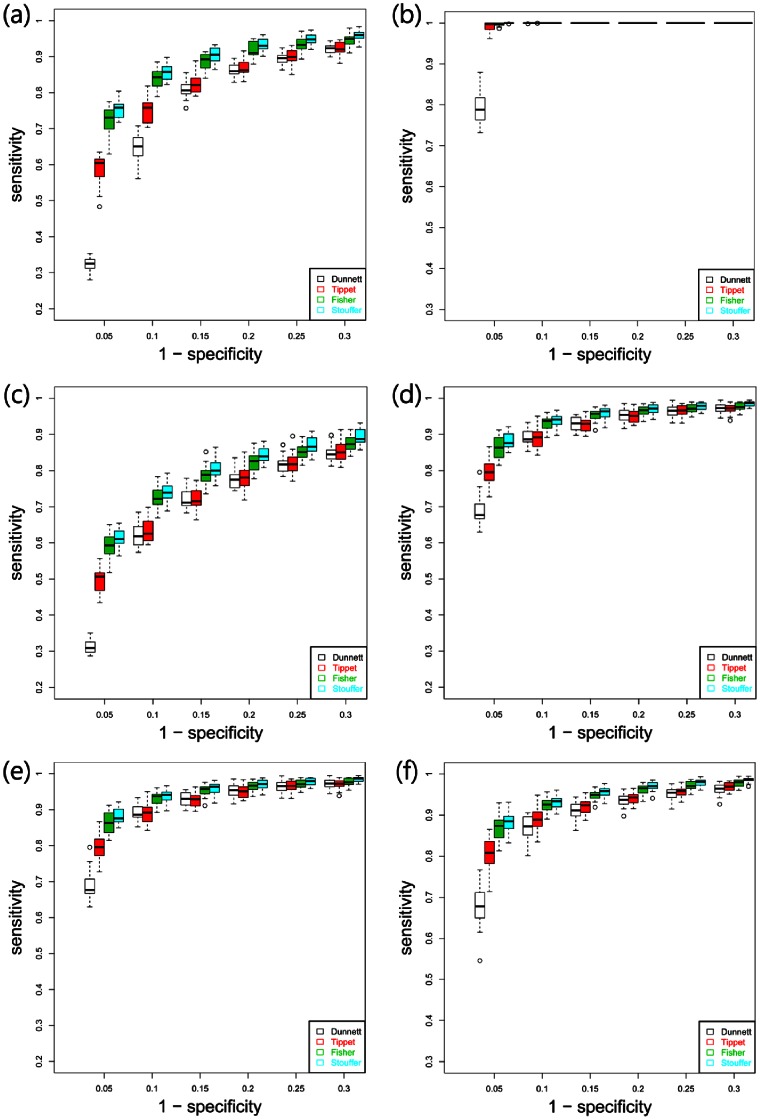
Boxplot of ROC curves for (A1) and (A2). (a, b) are for the standard normal distribution; (c, d) are for the t-distribution with degrees of freedom 5; and (e, f) are for the gamma distribution with parameters 3 and 1. Here, the number of samples in (a), (c), (e) are 5, and the number of samples in (b), (d), (f) are 20.

Figure 3 compared the receiver operating characteristic curves (ROCs) of three methods, 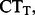 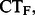 and 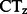
for each error distribution. Figure 3 (a, b) are for the standard normal distribution; Figure 3 (c, d) are for the t-distribution with degrees of freedom 5; and Figure 3 (e, f) are for the gamma distribution with parameters 3 and 1. Here, the number of replicates (k in the model above) in (a), (c), (e) is 5, and the number of replicates in (b), (d), (f) is 20.

The ROC curve plots two accuracy measures, false positive rate (FPR) and true positive rate (TPR), of a test, where the FPR is the probability that the test mistakenly detects the EEGs as DEGs, and the TPR is the probability that it correctly detects the DEGs. In each data set, we estimated their probabilities as portion
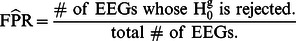
and



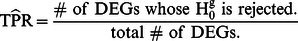
with various critical values for tests. The plot is the average of 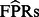 and 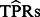 over 20 data sets simulated from the model described above. It shows that Stouffer’s test has the highest statistical power (specificity) in detecting differentially expressed genes, whereas Tippett’s procedure performs worst among three procedures based on p-values or z-scores. In addition, the computational burden of 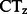 was much lighter than 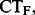 which relied on an additional re-sampling procedure. Dunnett’s test performs worse than all others in overall.

Table 2 summarized rank statistics of 200 DEGs among 4000 genes. In the table, we reported the averages of the quantiles of ranks of 200 DEGs (among 4000 genes) over 20 simulated data sets. Lower ranks of DEGs tells the higher detectability of DEGs by a given procedure.

**Table 2:** Summarized rank statistics of 200 DEGs among 4000 genes (A1 and A2).

		N = 5									
		Mean					SD				
		1st Qu.	Median	Mean	3rd Qu.	Max	1st Qu.	Median	Mean	3rd Qu.	Max
Normal	Dunnett	82.24	221.18	443.16	558.56	3589.85	10.32	14.42	25.12	43.52	324.07
	Fisher	59.03	145.21	331.24	356.42	3384.30	3.68	10.88	35.32	46.79	472.84
	Tippett	83.83	217.17	435.04	525.01	3391.70	8.28	17.99	39.80	60.59	384.98
	Stouffer	58.36	142.98	297.16	333.36	3203.95	3.53	11.90	29.57	37.66	669.17
T	Dunnett	94.70	284.28	638.28	804.97	3761.55	11.40	32.92	52.56	100.11	99.21
	Fisher	69.02	210.02	540.59	600.76	3895.35	6.30	22.12	50.20	87.10	141.64
	Tippett	96.59	286.72	630.83	813.27	3909.70	8.47	25.55	51.59	101.63	118.24
	Stouffer	66.00	195.83	491.50	546.64	3858.45	5.52	18.22	44.13	77.75	113.96
Gamma	Dunnett	201.17	689.35	1137.75	1873.45	3797.55	36.32	93.63	67.96	168.90	10.00
	Fisher	125.26	533.98	1025.33	1666.00	3954.50	20.07	65.32	69.14	160.15	44.74
	Tippett	212.86	724.78	1135.55	1812.45	3947.35	32.05	96.81	70.72	147.74	45.10
	Stouffer	113.16	460.48	911.77	1407.05	3939.35	14.60	52.96	62.11	125.25	94.28
		**N = 20**									
		**Mean**					**SD**				
		**1st Qu.**	**Median**	**Mean**	**3rd Qu.**	**Max**	**1st Qu.**	**Median**	**Mean**	**3rd Qu.**	**Max**
Normal	Dunnett	50.75	100.55	101.86	150.35	286.90	0.00	0.22	0.64	0.37	74.54
	Fisher	94.10	94.10	101.23	94.10	230.63	1.97	1.97	0.59	1.97	51.43
	Tippett	78.73	78.73	102.55	95.50	276.10	3.85	3.85	0.91	32.08	77.43
	Stouffer	50.75	100.50	100.79	150.25	227.20	0.00	0.00	0.32	0.22	41.81
T	Dunnett	54.23	122.70	251.45	268.41	2953.50	1.79	8.10	32.92	33.16	622.52
	Fisher	44.60	106.39	205.35	200.29	3024.65	13.69	3.55	24.06	16.08	628.35
	Tippett	52.24	119.87	251.48	264.62	3035.30	3.14	4.46	31.48	24.51	620.30
	Stouffer	51.24	106.13	189.69	195.46	2540.95	0.74	2.62	18.38	15.17	619.45
Gamma	Dunnett	51.88	117.38	271.52	274.95	3206.30	1.13	4.95	32.57	41.75	679.76
	Fisher	31.28	105.19	205.27	193.93	2919.50	2.95	2.97	22.90	17.40	614.13
	Tippett	52.60	117.45	255.89	262.48	3021.90	2.32	6.03	29.03	42.12	459.53
	Stouffer	50.95	104.03	189.85	187.89	2540.40	0.52	2.30	20.88	14.95	769.03

Table 3 summarized the true FDR from 20 data sets. For each simulated data set, the FDR was controlled using q-value by Storey [20]) and the true FDR was evaluated (in the simulated data set, we have a list of 200 DEGs). When sample size is small (N = 5), the FDRs are slightly larger than the targetted FDR in all procedures. However, as the sample size increases (N = 20), the FDR is better controlled at the targetted level (0.1 or 0.2 in the table). In particular, the FDR levels of Tippett’s test and Fisher’s omnibus test are close to the aimed level, whereas the Stouffer’s test gives the FDR value lower than the target. This indicates that the proposed procedure using Stouffer’s test is rather conservative in detecting DEGs. It is reassured from the data example in next section.

**Table 3:** Summary of FDR estimates based on 20 data sets having 200 DEGs among 4000 genes (A1 and A2).

			FDR = 0.1	FDR = 0.2
Normal	N = 5	Dunnett	0.61 (0.02)	0.69 (0.02)
		Fisher	0.10 (0.06)	0.22 (0.05)
		Tippett	0.17 (0.20)	0.22 (0.11)
		Stouffer	0.03 (0.08)	0.11 (0.06)
	N = 20	Dunnett	0.18 (0.03)	0.31 (0.03)
		Fisher	0.11 (0.02)	0.21 (0.03)
		Tippett	0.11 (0.02)	0.22 (0.03)
		Stouffer	0.01 (0.01)	0.02 (0.01)
T	N = 5	Dunnett	0.59 (0.03)	0.68 (0.02)
		Fisher	0.14 (0.09)	0.22 (0.08)
		Tippett	0.20 (0.19)	0.24 (0.11)
		Stouffer	0.03 (0.07)	0.07 (0.08)
	N = 20	Dunnett	0.18 (0.03)	0.31 (0.03)
		Fisher	0.10 (0.02)	0.20 (0.04)
		Tippett	0.10 (0.02)	0.19 (0.03)
		Stouffer	0.04 (0.02)	0.09 (0.02)
Gamma	N = 5	Dunnett	0.74 (0.03)	0.78 (0.02)
		Fisher	0.22 (0.23)	0.26 (0.12)
		Tippett	0.55 (0.39)	0.49 (0.36)
		Stouffer	0.33 (0.41)	0.13 (0.12)
	N = 20	Dunnett	0.18 (0.03)	0.30 (0.05)
		Fisher	0.10 (0.03)	0.19 (0.04)
		Tippett	0.10 (0.03)	0.21 (0.03)
		Stouffer	0.04 (0.02)	0.09 (0.03)

### Data Example

We analyzed the microarray data described in the experimental section. The data sets are available from GEO (Gene Expression Omnibus; http://www.ncbi.nlm.nih.gov/geo/query/acc.cgi?acc=GSE31307). The data aimed to understand genotoxicity and carcinogenicity of compounds in view of systems toxicology. In the microarray data, 2-AAF, 3′MeDAB and DEN were known as genotoxic carcinogens, and clofibrate, ethionine and 1,4-dioxane were known as non-genotoxic carcinogens.

We preprocessed the data before the analysis using the RMA (Robust Multi-array Average) method. The RMA method was developed by Bolstad et al. (2003) [21] for the normalization of Affymetrix GeneChip array data. The RMA method is a three-step procedure: (1) Background correction and data transformation; (2) Normalization at the probe level using the quantile method; and (3) Model parameter adjustment to get normalized expression levels. The RMA method is freely available from the bioconductor website (http://bioconductor.org).

We found the DEGs from the control in non-genotoxic and genotoxic carcinogens. In each of non-genotoxic and genotoxic carcinogens, we assumed that 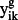 for 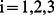 had the common mean 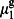 whereas 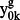 had the mean 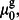 for the 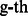 gene. We tested
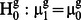
using 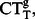 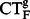 and 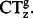 The p-values of 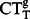 and 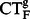 were approximated by the permutation method with 5,000 permutation samples. On the other hand, the p-value of 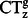 was computed as



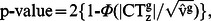
where



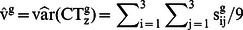
and 
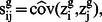
which was estimated as the sample covariance of 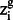
and 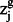
over 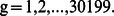 The DEGs were selected to make Type I error be smaller than 0.05 in all cases.

**Figure 4:**
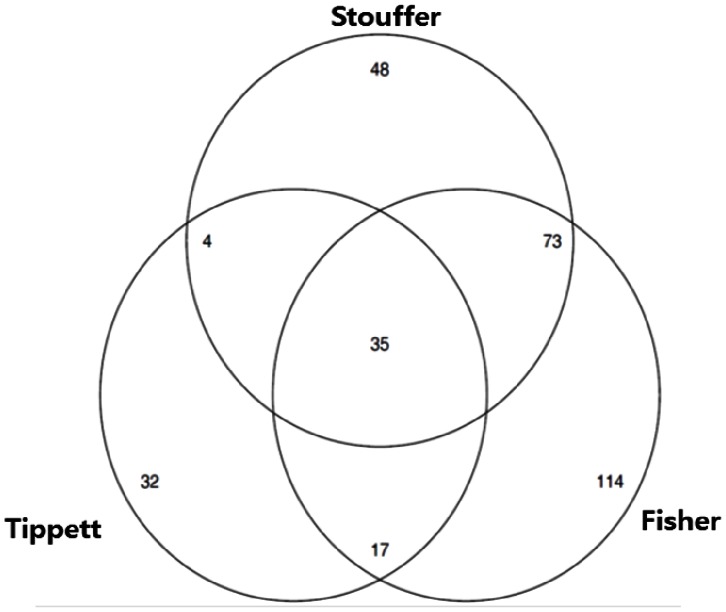
Venn diagram of selected DEGs of genotoxic carcinogen using three methods (Tippett, Fisher, Stouffer) α = 0.001.

**Figure 5:**
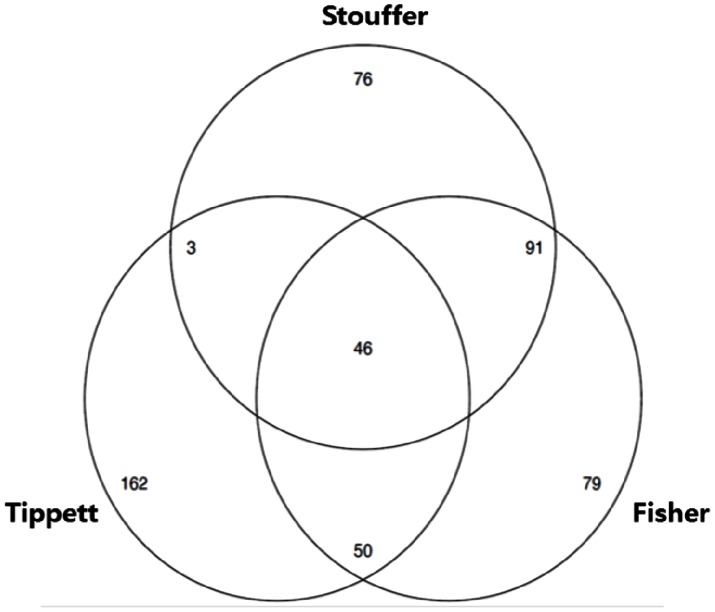
Venn diagram of selected DEGs of non-genotoxic carcinogen using three methods (Tippett, Fisher, Stouffer) α = 0.001.

Figures 4 and 5 are Venn diagrams showing how the selected genes were distributed in the three methods. Figure 4 is the plot for the genotoxic carcinogens and Figure 5 is that for non-genotoxic carcinogens. The figures showed that the selected genes by z-score method were more similar to those by Fisher’s method than by Tippett’s method. This would not be unexpected as it can be seen in both 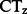 and 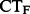 averaged outcomes of three partial tests whereas 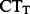 took the minimum of them.

The number of selected genes is summarized in Table 4.

**Table 4:** The number of selected genes.

Method	Tippett		Fisher		Stouffer	
	G	NG	G	NG	G	NG
α = 0.010	1984	1046	1611	1623	1316	1202
α = 0.001	261	88	266	239	216	160
FDR = 0.05	209	12	266	137	5	2

In the table, α implies the significant level of the test. “G” and “NG” imply genotoxic compound and non-genotoxic compound, respectively. The genes were selected at the significance level 0.001.

The genes, which were obtained through three different methods, were interpreted based on DAVID gene ontology database. In case of Tippett’s method, between 261 DEGs (α = 0.001) of genotoxic carcinogen, 10 genes such as Opa1, AIMP, YWHAB, etc., were considered as marker candidates among 259 genes whose expression was altered in the presence of genotoxic carcinogen. The other two genes were excluded as they revealed the same response when the non-genotoxic carcinogen administered. Similarly, 12 genes such as Stk3, CTNNBL1, PPP1F15A, etc., were selected among 266 genes as promising marker candidates by Fisher’s method and 17 genes such as PPP2R2B, tnfrsf11b, acvr1, etc., were selected among 216 genes by Stouffer’s method. Consequently, the proportion of marker candidates in selected genes obtained by Stouffer’s method was the largest among those three methods. In particular, 9 genes such as pawr, Opa1, STK17B, AEN, FASTKD3, YWHAB, Stk3, ALS2 and CIDEC demonstrated high significance consistently in more than two different methods.

Through screening by Tippett’s method, 88 DEGs (α = 0.001) of non-genotoxic carcinogen were chosen. Among 86 genes, whose expression was transformed in the presence of non-genotoxic carcinogen within 88 genes, 15 genes such as snai1, mgea5, Pcsk6, etc., were further selected as marker candidates according to their mechanisms. In addition, 25 genes such as EDNRA, Pgcp, ABHD2, etc., were chosen as potent maker candidates among 239 genes by Fisher’s method and 28 genes such as MTDH, GCH1, ADH4, etc., were selected among 160 genes by Stouffer’s method. As a result, the proportion of marker candidates in selected genes obtained by Stouffer’s method was the highest among those three methods. Remarkably, 6 genes such as Tsg101, EDNRA1, EDNRA2 EDENRA3, PRKAR1A and HDAC2 are the more valuable markers of non-genotoxic carcinogen as they demonstrated high significance consistently in more than two different methods.

**Figure 6:**
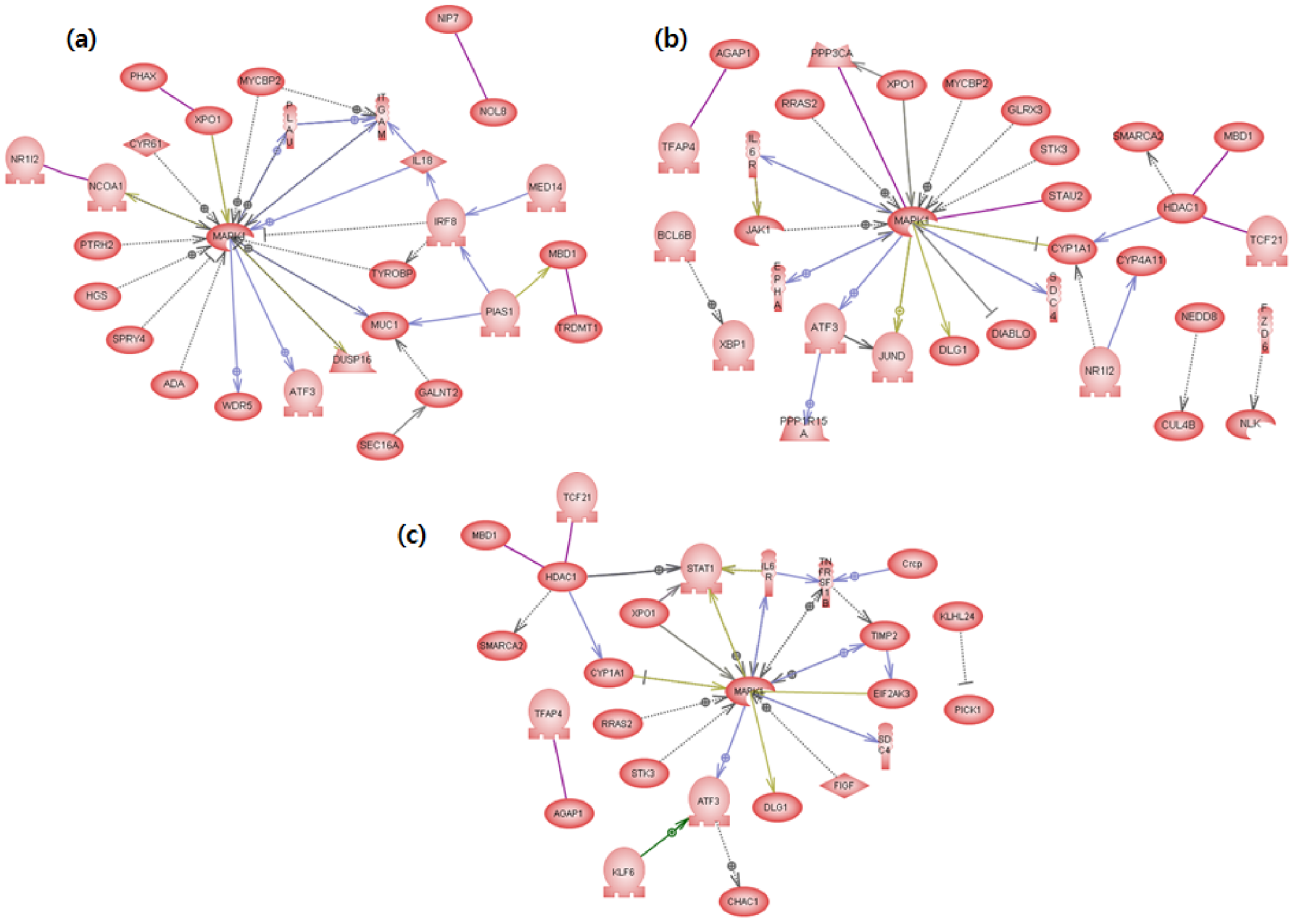
The genes that show significant differences in expression between control and genotoxic carcinogens. Each entity represents protein transcribed from selected DEGs and an arrow indicates a connection: (a) Tippett, (b) Fisher, (c) Stouffer.

**Figure 7:**
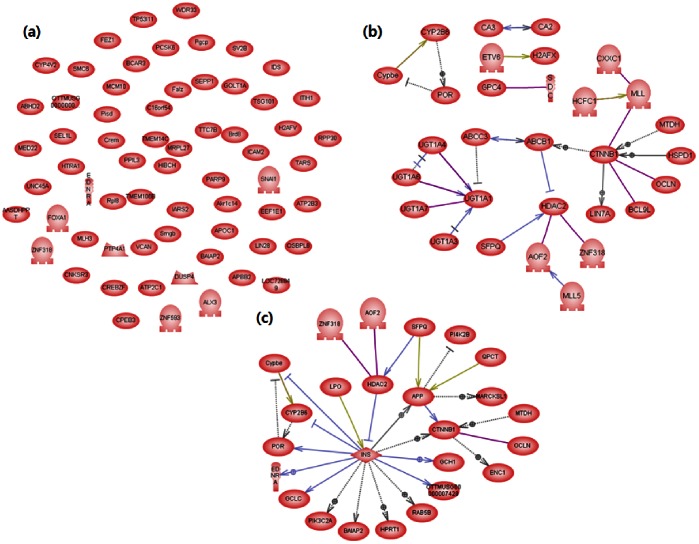
The genes that show significant differences in expression between control and non-genotoxic carcinogens. Each entity represents protein transcribed from selected DEGs and an arrow indicates a connection: (a) Tippett, (b) Fisher, (c) Stouffer.

Pathway analysis was conducted on selected genes. It finds relationship between the genes and shows why genes have become differentially expressed genes (DEGs). Figures 6 and 7 are demonstrations of search for all relations registered in the database on the subject, the six DEG lists, which were the search result of pathway analysis, and expressed them as a pathway map. Figure 6 is a result of pathway analysis on the genes whose properties were significantly changed in the control group and genotoxic carcinogen-treated group, and is expressed in the order of (a) Tippett, (b) Fisher, and (c) Stouffer. As each map showed a similar level of networking, we discovered that the three methods were similarly efficient when searching for genes whose properties changed at the time of injecting genotoxic carcinogen. This can be interpreted as the chemical structure of genotoxic carcinogen having high affinity for DNA and damaging the genome in a short time, so that the range of changing gene properties is wide and the sorting process is relatively easy. On the other hand, in Figure 7, which compares non-genotoxic carcinogen and control, the map for (a) was not formed. Fisher’s method (b) was also insufficient to find the largest part of a network.

The validity of the selected gene list is verifiable through the contents of the gene. In Figure 6, the center gene of the network was mapk1 in all results upon the injection of genotoxic carcinogen. This result matched with prior findings of existing research, because mapk1 is a major element for the Ras-MAPK network, a representative mechanism for cell proliferation signal transduction. The signal mechanism of Ras-MAPK, a representative path of proliferation stimulation, reveals a network to determine whether the cell should proliferate, stop proliferating, or die; this signaling mechanism is a core issue in the research on cancer. Therefore, the Ras-MAPK mechanism can be an indicator to examine the genotoxicity of chemicals. The results showed that mapk1 was included in the map upon genotoxic carcinogen injection as shown in Figure 6 (a) (b) (c), but it was not included upon non-genotoxic carcinogen injection as shown in Figure 7 (a) (b) (c). Thus, all of the analysis methods showed different efficiencies, but the direction chosen by each was reasonable.

### Conclusions

In this paper, we revisited Stouffer’s method based on z-scores and showed that it was useful for large-scale microarray data analysis. In particular, when we combined dependent partial test results, unlike that in other popular methods such as Tippett’s test and Fisher’s omnibus test, the null distribution of the combined statistic in Stouffer’s test could be easily estimated from the data and did not require any additional numerical procedure. The numerical study showed that Stouffer’s test had higher true positive rates (TPR) than Tippett’s method and Fisher’s method when we tested a large number of hypotheses simultaneously. In addition, real data analysis also showed the advantage of Stouffer’s method over the others.

We conclude the paper with brief discussions on several issues, which are not fully covered in the main text.

First, in both numerical study and data analysis, the false discovery rate (FDR) was estimated using the q-value method by Storey (2002) [20]. We found that the q-value method with the Stouffer’s test often overestimated the FDR and provided a *conservative* list of DEGs; it only detected a smaller number of genes, which were much differentially expressed, than other methods. This phenomenon is from deviations between theoretical (which is used in this paper) and empirical distribution of test statistics. One simple remedy for this would be the empirical Bayes (EB) procedures by Efron (2007) [22], Schwartzman et al. (2008) [23], and many others. They estimate the null distribution of testing statistics from data, and they are robust to any deviation from theoretical assumption.

Second, the reviewer points out difficulty from the dependence of expression levels among genes. As discussed in Efron (2007) [22], it is one source of deviation between theoretical and empirical distribution of test statistics (not only 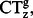 but also 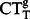 and 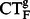). Again, the EB procedures would be a good remedy for this difficulty.

Finally, in recent, Benjamini and Heller (2008) [24] introduce partial conjunction hypothesis on the number of false null hypothesis, which is much general than the complex hypothesis considered in this paper. They propose a procedure to combine p-values (of partial tests) to test the hypothesis, and it would be interesting to investigate the advantages of using z-scores over p-values.
